# A sex and gender perspective for neglected zoonotic diseases

**DOI:** 10.3389/fmicb.2022.1031683

**Published:** 2022-10-20

**Authors:** Daniela Fusco, Guillermo Z. Martínez-Pérez, Aaron Remkes, Alessandra Mistral De Pascali, Margherita Ortalli, Stefania Varani, Alessandra Scagliarini

**Affiliations:** ^1^Department of Infectious Diseases Epidemiology, Bernhard Nocht Institute for Tropical Medicine, Hamburg, Germany; ^2^German Center for Infection Research (DZIF), Hamburg, Germany; ^3^African Women's Research Observatory (AfWORO), Barcelona, Spain; ^4^Section of Microbiology, Department of Experimental, Diagnostic and Specialty Medicine, Alma Mater Studiorum University of Bologna, Bologna, Italy; ^5^Microbiology Unit, IRCCS Azienda Ospedaliero-Universitaria di Bologna, Bologna, Italy

**Keywords:** neglected zoonotic diseases, gender, sex, schistosomiasis, leishmaniasis, monkeypox

## Introduction

Individuals are affected differently by infectious diseases depending on their sex and gender identity, as well as the gender- and sex-specific values and norms held by society and healthcare providers (Ingersoll, 2017; Scully, 2022).

Among all the infectious diseases, neglected tropical diseases (NTDs) is a group of diseases of poverty, prevalent in tropical areas but not limited to them (Hotez, 2021; Molyneux et al., 2021). The acronym NTD suggests that these are diseases of populations living in the tropics, leading to a scarcity of data, treatment and diagnostic solutions. However, NTDs are also present among the socioeconomically-disadvantaged populations living in non-tropical regions, such as Europe and North America (Hotez and Gurwith, 2011). Most NTDs are zoonoses, i.e., infections transmitted from animals to humans and vice versa, which contributes to the complex epidemiology of these diseases.

Zoonotic NTDs are defined as neglected zoonotic diseases (NZDs) (King, 2011). The impact of sex and gender in populations affected by NZDs has been scarcely explored, but is imperative for their effective control, as NZDs can in most instances be prevented and controlled by public health interventions (King, 2011). A stronger emphasis on the integration of sex and gender perspectives in NZD research could help stakeholders and policy makers to conceptualize transformative solutions for most burdened populations.

The term sex refers to biological differences between males, females, and intersex persons. Sex-specific anatomical-physiological and hormonal differences influence the course of infectious diseases (World Health Organization, 2007). Studies have highlighted that hormonal and chromosomal factors contribute to sex-specific differences in host responses to some infections. For example, the ability of a parasite to affect female or male individuals differently may be due to the regulation of the immune response by sex hormones (Nava-Castro et al., 2012).

On the other hand, the term gender refers to the norms, values, functions, responsibilities or expectations that society imposes on people according to their assigned sex at birth (Connell, 2021). As gender affects individuals' cosmovision of health and disease, NZD research needs to take gender into account since it impacts demand, access and utilization of NZD prevention and control. In addition, a One Health perspective should be adopted as NZDs occur at human/animal/environment interfaces with different gender-related exposure risks.

This opinion paper argues that considering sex- and gender-based differences and commonalities is crucial in the prevention and control of NZDs. Mainstreaming sex and gendered perspectives can guide research, clinical management and public health strategies that pursue the achievement of the Sustainable Development Goal 3: Good Health and Wellbeing for all, regardless of peoples' sex and gender (Manandhar et al., 2018).

In this paper, we will examine schistosomiasis, leishmaniasis and monkeypox as case studies where a sex- and gender-transformative approach could reduce the burden of these diseases.

## Sex and gender barriers in the control of schistosomiasis

Human schistosomiasis is a parasitic disease with a zoonotic intermediate host, the freshwater snail, and is mainly caused by the species *Schistosoma mansoni* and *S. haematobium*, which infect humans through contact with contaminated water (McManus et al., 2018). The most typical chronic manifestations of schistosomiasis are hepatic fibrosis and urogenital schistosomiasis (McManus et al., 2018).

From a sex perspective, schistosomiasis infections cause a stronger pro-inflammatory response in men (Klein, 2004; Schneider-Crease et al., 2021). From a gender perspective, research suggests that men may be at a higher risk of contracting schistosomiasis due to their more frequent engagement, in comparison to women, in occupations that involve contact with contaminated waters (Ayabina et al., 2021). However, the occupational risk also exists for women living in high-burdened schistosomiasis environments, as they come into contact with water for household-related activities and fishing practices not often associated to women (Standley et al., 2011; Munisi et al., 2016; Sevilimedu et al., 2017; Pouramin et al., 2020; Ayabina et al., 2021).

Genital schistosomiasis is a chronic manifestation of the disease that affects all sexes (Christinet et al., 2016). Chronic infections with *S. haematobium* can lead to genital lesions, damage of reproductive organs and infertility (Kayuni et al., 2019). While sex-disaggregated data exists describing the burden of disease, the imbalanced number of studies on Male Genital Schistosomiasis (MGS), compared to those on Female Genital Schistosomiasis (FGS) makes it difficult to estimate whether more males than females are affected by the disease (Kayuni et al., 2019; Bustinduy et al., 2022). Task forces have been established to study and implement health programs aimed at managing FGS while MGS has received little attention (Bustinduy et al., 2022; Schistosomiasis. COR-NTD. 2022, 2022).

However, certain societal considerations of infertility as a community and family problem have a more detrimental effect on women than on men (Allotey and Gyapong, 2005; Downs et al., 2011; Hotez and Whitham, 2014; Corno et al., 2020; Engels et al., 2020). In addition, the widespread belief in sub-Saharan regions that FGS is sexually transmitted is a major barrier to seeking treatment (Downs et al., 2011; Corno et al., 2020; Engels et al., 2020). Public perceptions of FGS can affect a woman's position in the family and community, leading to her exclusion from traditional social circles and, in the worst cases, gender-based violence (Allotey and Gyapong, 2005; Corno et al., 2020; Engels et al., 2020). On the other hand, haematuria, a sign of urogenital schistosomiasis, is often perceived as a sign of puberty and virility in boys (Vlassoff and Bonilla, 1994; Kukula et al., 2019; Ayabina et al., 2021).

## Sex and gender perspectives in human leishmaniasis

Human leishmaniasis is a sandfly-borne disease caused by different protozoan species of the genus *Leishmania* (World Health Organization, 2010). Leishmaniasis occurs in two principal clinical forms: visceral leishmaniasis (VL) and cutaneous leishmaniasis (CL). There is evidence that both VL and CL are more prevalent in males than in females (World Health Organization, 2010).

The role of sex hormones in influencing the infection level and disease severity has been extensively demonstrated in animal studies, and there exists some evidence for this in humans as well (de Araújo Albuquerque et al., 2021).

From a gender perspective, no gender-related differences in the prevalence of asymptomatic *Leishmania* infection could be described in southern Europe (Ibarra-Meneses et al., 2019; Ortalli et al., 2020). In contrast, studies in sub-Saharan Africa suggest that leishmaniasis may exert a higher burden in men (World Health Organization, 2010). Some researchers have considered that, due to their frequent engagement in outdoor activities, men may be considered at higher risk of exposure to infected insects and thus at higher risk of developing leishmaniasis than women (World Health Organization, 2010). In societies where gendered values put more pressure on women than on men to meet certain beauty standards, CL imposes a greater disease burden on women. CL is a disfiguring disease that often leaves scars on visible body parts (Bilgic-Temel et al., 2019). In countries such as Pakistan or Afghanistan, it has been reported that facial lesions can lead to self-isolation, and to exclusion of women from all aspects of life (Bennis et al., 2018).

The existence of disaggregated data does not provide accurate information on the sex distribution of the disease. In many endemic areas, access to health care services for women is poor, leading to possible underreporting of leishmaniases cases among females (World Health Organization, 2010). Unsurprisingly, a recent meta-analysis showed that two-thirds of the participants enrolled in clinical trials on VL conducted in the past 40 years were adult males (Dahal et al., 2021).

## The changing exposure pattern of monkeypox from a gender perspective

Monkeypox is caused by the *Monkeypox virus* (MPXV) an epitheliotropic orthopoxvirus, endemic in western and central Africa (Essbauer et al., 2009; Tack and Reynolds, 2011). The disease has recently attracted worldwide attention as a growing number of cases is being reported in Europe and North America (The Lancet Infectious Diseases, 2022).

The sex and gender dimensions of this infection are poorly explored. There is a dearth of studies with meaningful data for developing sex- and gender-transformative strategies, guidelines or recommendations for the prevention and control of the spread of MPXV. As an example, from a gender perspective, a study conducted in the Democratic Republic of Congo reported that men appear to be most often affected due to the higher risk of exposure to animals associated with the occupational practices they most engage in (hunting, skinning, and trading), while women are mainly exposed due to domestic duties such as contact with contaminated meat or MPXV-infected family members (Whitehouse et al., 2021). Further studies following Whitehouse et al. (2021), are warranted in different geographies to avoid making unfounded generalizations about the reduced or increased risk of MPXV acquisition.

The 2022 monkeypox outbreak in non-endemic countries is showing novel epidemiological features compared to endemic areas. A recent series of cases suggested that the outbreak was disproportionally affecting white gay and bisexual men in the Northern Hemisphere (Thornhill et al., 2022). As a result of these first publications mass media and public health institutions amplified the notion that MPXV may be acquired through sexual networks (Simões and Bhagani, 2022). On July 2022, the World Health Organization (WHO) declared the global monkeypox outbreak a public health emergency of international concern, and called for prevention strategies amongst gay and bisexual men (Taylor, 2022). The initial reactions by the media and WHO, among other stakeholders, have been received with criticism. The scientific community is wary of these proposed strategies and is conceptualizing alternatives to raise public awareness about monkeypox (Kupferschmidt, 2022). Learning from the experiences of the HIV/AIDS pandemic, gender-blind communication strategies could fuel stigma against LGBTQIA+ communities and create a false risk perception among other non-LGBTQIA+ groups (UNAIDS, 2022).

## Discussion

Gender and sex are two different but interweaved entities that need to be considered in every health context, as they clearly affect the potential for infection and the course and severity of infectious diseases (Gay et al., 2021). Evidence-based data is urgently needed to apply a gender- and sex-transformative approach to NZDs prevention and control. However, there is paucity of data on infections causing NZDs, which tend to affect the most vulnerable populations.

This opinion paper focuses on three NZDs, namely schistosomiasis, leishmaniasis and monkeypox, to highlight their relevance in applying a sex and gender perspective to their prevention and control.

Schistosomiasis is a clear example of the existence of sex and gender differences in the manifestation, perception and management of the disease. An example of this is the occupational risk of contracting schistosomiasis, which is generally treated as a men's issue, whereas if a gender perspective was applied, one could assume that women and men are proportionally exposed because of their occupations. On the other hand, genital schistosomiasis is mostly researched as a female disease, although it also affects males.

Studies on leishmaniasis show that from both a sex and gender perspective, males seem more affected than females. Nevertheless, most of the sex data are based on studies enrolling for the most part males (Dahal et al., 2021). Additionally, when addressing the risk of leishmaniasis in the Northern Hemisphere (Ibarra-Meneses et al., 2019; Ortalli et al., 2020) it appears that gender does not play a decisive role in influencing the rate of infection, which could be explained by the different social norms across countries.

In the case of both schistosomiasis and leishmaniasis, it is noteworthy that despite the reported higher risk in men, these diseases place a heavier societal burden on women. The extent of this burden has not been precisely quantified yet. Hence, greater attention from donors and stakeholders in health research should be given to women and gendered minorities in their efforts to addressing the impact of these NZDs.

Monkeypox is an NZD largely ignored in the past (Alakunle and Okeke, 2022). The recent outbreak in non-endemic countries has brought unprecedent attention to the disease. In non-endemic countries, the higher infection rate observed at the start of the global outbreak within the gay and bisexual men, led to the recommendation to implement prevention and screening strategies targeting these specific “communities.” However, MPXV is a zoonosis. Hence, the role of animals and the possibility that MPXV might be affecting individuals regardless of their sexual orientation, should not be overlooked (Haider et al., 2022).

By examining these three NZDs, we identified theoretical and methodological gaps in addressing sex and gender differences. There is a need to apply a holistic methodology combining sex- and gender-oriented approaches with a One Health approach to gain proper insights and tackle NZDs at their sources. Research tools capable of capturing gender-based nuances need to be developed and made available. Medical education should adapt programmes and courses that put these diseases into a gender perspective. Adopting the idea of gender-specific solutions for the management of NZDs may promote a more equitable, safe and effective health care for all. A gender-oriented mindset needs to be also mainstreamed into decision-making to encourage respect and consideration of the diversity of sex and gender identities without promoting stigma (Lancet, 2019).

In conclusion, a sex and gender lens in NZD research can help to understand whether and under what circumstances individuals of any sex or gender identity suffer a greater share of the NZD burden, due to a range of bio-anatomical factors that may act in conjunction with the disproportionate poverty, illiteracy, violence, food insecurity and higher risk of animal-borne pathogen transmission, which affect middle-low-income countries as well as socially-disadvantaged groups in high-income countries. Ultimately, it can maximize the impact of public health interventions and strategies aimed at eliminating some of these diseases by 2030.

## Author contributions

DF, SV, and AS contributed to the conceptualization of the manuscript. DF and GZM-P contributed to framing the structure of the manuscript. DF and AR were responsible of the schistosomiasis contribution. SV and MO were responsible of the leishmaniasis contribution. AS and AMDP were responsible of the monkeypox contribution. All authors revised and approved the manuscript before submission.

## Funding

This work was partly funded by the German Center for Infection Research (DZIF) through the projects NAMASTE (project number: 8008803819) and VAMS (project number: TI 07.003).

## Conflict of interest

The authors declare that the research was conducted in the absence of any commercial or financial relationships that could be construed as a potential conflict of interest.

## Publisher's note

All claims expressed in this article are solely those of the authors and do not necessarily represent those of their affiliated organizations, or those of the publisher, the editors and the reviewers. Any product that may be evaluated in this article, or claim that may be made by its manufacturer, is not guaranteed or endorsed by the publisher.
